# Clinical and economic analysis of Gastrodin injection for dizziness or vertigo: a retrospective cohort study based on electronic health records in China

**DOI:** 10.1186/s13020-021-00561-9

**Published:** 2022-01-04

**Authors:** Yunfeng Lai, Ruoning Wang, Wei Li, He Zhu, Shuyang Fei, Honghao Shi, Nan Lu, Carolina Oi Lam Ung, Hao Hu, Sheng Han

**Affiliations:** 1 School of Public Health and Management, Guangzhou University of Chinese Medicine, Guangzhou, China; 2 State Key Laboratory of Quality Research in Chinese Medicine, Institute of Chinese Medical Sciences, University of Macau, Taipa, Macao SAR, China; 3 Department of Continuing Medical Education, Peking University Health Science Center, Beijing, China; 4 Department of Real-World Evidence and Pharmacoeconomics, International Research Center for Medicinal Administration, Peking University, Beijing, China; 5 Department of Vasculocardiology, AnZhen Hospital, Affiliated to Capital Medical University, Beijing, China; 6 Inchuan Medlinker Internet Hospital, Yinchuan, NingXia Hui Autonomous Region China

**Keywords:** Gastrodin injection, Dizziness, Vertigo, Extract of Ginkgo Biloba Leaves injection, Electronic health record

## Abstract

**Background:**

Dizziness and vertigo are common clinical symptoms. Gastrodin injection has shown clinical effects on dizziness or vertigo. However, little is known about the effectiveness and costs of combining Gastrodin injection with conventional treatment on dizziness or vertigo in daily practice. This study aimed to analyze the clinical and economic effects of Gastrodin injection for patients with dizziness or vertigo in comparison to Extract of Ginkgo Biloba Leaves injection in real-world practice.

**Methods:**

Data was collected from the Hospital Information System of 131 hospitals across China from January to December 2018. Patients whose primary discharge diagnosis was dizziness or vertigo according to ICD-10 diagnostic coding were included and divided into two samples: sample of dizziness or vertigo; sample of dizziness or vertigo, with the complication of cerebral infarction. Comparative analysis of the medical cost per hospitalization, hospitalization duration, effective rates, and cure rates between the group of Gastrodin injection and the group of Extract of Ginkgo Biloba Leaves injection was conducted. Propensity Score Matching was used to control potential confounding factors.

**Results:**

In the sample of dizziness or vertigo, although there was no significant differences on hospitalization duration (*P* = 0.080), the group of Gastrodin injection was significantly better than the group of Extract of Ginkgo Biloba Leaves injection (*P* < 0.001) in terms of treatment effect and the per capita hospitalization cost. In the sample of dizziness or vertigo, with the complication of cerebral infarction, there was no significant difference (*P* = 0.371) in terms of hospitalization duration, but the group of Gastrodin injection was significantly better than the group of Extract of Ginkgo Biloba Leaves injection (*P* = 0.009) in terms of treatment effect, and significant difference regarding the per capita hospitalization cost (*P* < 0.001).

**Conclusions:**

Gastrodin injection showed advantages for inpatients with dizziness or vertigo compared with Extract of Ginkgo Biloba Leaves injection. Future studies using prospective pragmatic controlled trials can test and explore more about the effects of Gastrodin injections on dizziness or vertigo.

**Supplementary Information:**

The online version contains supplementary material available at 10.1186/s13020-021-00561-9.

## Background

Dizziness is divided into vertigo, presyncope, disequilibrium, and lightheadedness in the world [1–3]. Dizziness and vertigo are the main manifestations of various central or peripheral vestibular syndromes caused by cerebrovascular disease. Dizziness and vertigo are common morbid syndrome, and the prevalence rate increases with age, which are the top three main reasons for the elderly to see a doctor. Patients are often complicated with various complications, rapid development of the disease, and hidden causes, which significantly increased clinical treatment difficulty [4]. According to the anatomic location of the lesions in clinical, vertigo can be divided into systemic vertigo caused by vestibular nervous system lesions and non-systemic vertigo caused by lesions outside the vestibular system [5]. The isolated dizziness or vertigo of cerebrovascular disease is easily misdiagnosed and might cause severe consequences [6]. However, dizziness is the most common clinical symptom in posterior circulation cerebral infarction with the highest incidence [7]. Some studies on the research between incidence rate of acute cerebrovascular disease and isolated dizziness or vertigo have shown that the probability of acute cerebrovascular disease (cerebral infarction or cerebral hemorrhage) with acute dizziness or vertigo, nystagmus and/or balance disorders is 4.6–11% [8, 9].

Specifically, stroke has become the most common high disability and deadly disease globally [10] and the leading cause of death in China in recent years [11]. The age of stroke onset was earlier than before, and the prevalence and hospitalization rate showed a significant upward trend [12]. Cerebral infarction accounts for about 70% of stroke, and posterior circulation cerebral infarction accounts for about 20% of ischemic stroke [13]. Therefore, dizziness and vertigo have caused a heavy economic burden on patients and the health care systems worldwide [14].

According to the Multidisciplinary Expert Consensus of Diagnosis and Treatment of Vertigo (2017 edition), routine treatment with western medicine with the anti-inflammatory action of glucocorticoid is the first choice thrombolysis, antiplatelet, and anticoagulation [15]. Gastrodin is a active component extracted from the traditional Chinese medicine Gastrodia elata Blume, called *Tianma* in Chinese [16]. Gastrodia elata Blume and Gastrodin have low toxicity [17] and have pharmacological activities such as dizziness, stoke, headache, sedative, analgesic and neurological disorder-improving [18–21], which have a potential role in the protection of nerve cells [22, 23].

Gastrodin injection is mainly used in the clinical treatment of dizziness, vertigo, stroke, insomnia, headache, neurasthenia, coronary heart disease, and epilepsy [24–26]. The clinical effect is positive, and there is no significant adverse reaction [27]. Compared with western medicine, Gastrodin injection has better effectiveness and safety in treating cerebral infarction [28]. Furthermore, based on a multi-center single-blind randomized controlled trial, Gastrodin injection is safe and effective for vertigo and is better than betahistine injection in relieving vertigo symptoms [29].

Although Gastrodin injection showed sound clinical effects in these studies, there is little known about the clinical and economic evaluations of Gastrodin injection in real-world practice. Some retrospective studies had compared Gastrodin injection with other drugs in the treatment of dizziness and vertigo [30–32]. However, the actual conditions of patients are evidence of clinical medication decisions by doctors. It must consider realistic comparator drugs to evaluate specific medicine products' clinical and economic effects [33]. The leveraging of real-world data can improve clinical evaluation and that is one of the priority areas for improving public safety and facilitating innovation in China [34]. From current literature, there is still lacking studies on the evaluation of curative effect and economic benefit of the Gastrodin injection with the comparison of other drugs for patients in the real clinical environment.

In clinical practice, Extract of Ginkgo Biloba Leaves injection has been widely used for dizziness, vertigo, and cerebral infarction with low toxicity and a good curative effect with little adverse reaction [35–37]. It is a naturally extracted medicine, like Gastrodin injection, which can provide new treatment with high efficiency and low toxicity at low development cost [38]. The development of phytomedicine based on modern medicine and pharmaceutical theory is an innovation and has been recognized [39]. Thus, the comparison between these two naturally extracted medicines is more convincing, and it also can provide practical references for guiding the practice of interchangeability of the same type of drugs.

Therefore, this study aimed to analyze the clinical and economic effect of Gastrodin injection for patients with dizziness or vertigo in comparison to Extract of Ginkgo Biloba Leaves injection in real-world practice. It's expected that the findings can provide references for clinicians to optimize treatment options.

## Methods

### Research design

This study was a retrospective cohort study to compare the clinical and economic effects of Gastrodin injection and Extract of Ginkgo Biloba Leaves injection for hospitalization patients diagnosed with dizziness or vertigo (see STROBE checklist in Additional file 1).

### Composition information

In 1980, the chemical synthesis of Gastrodin was completed. The State Medicine Administrative Bureau approved Gastrodin injection of China as a new drug and first authorized the KPC Pharmaceuticals, Inc to manufacture in 1984. At present, 25 pharmaceutical companies are producing Gastrodin injection, with 37 approvals (including different specifications).

### Botanical

Gastrodia elata Blume (Orchidaceae) is an obligate mycoheterotrophic plant, an orchid popularly used in traditional Chinese medicine [40]. It is primarily found in China, Nepal, Bhutan, India, Japan, North Korea, Siberia, and Taiwan [41, 42]. In China, it grows mainly in Sichuan, Yunnan, Guizhou, and Hunan Province [43], where can provide high-quality daodi medicinal products [44]. Gastrodia elata Blume is cited by the Pharmacopoeia of China [45] and has been used in oriental medicine in East Asia to treat various diseases, including neurological disorders as an anticonvulsant, analgesic, and sedative medication [46].

### Chemical

Gastrodin injection is a kind of traditional Chinese medicine injection that is extracted and refined from G. elate [28]. The main ingredient is gastrodin and identified by the State Medicine Administrative Bureau of China. Since the early 1950s, China has begun to study the chemical composition of gastrodin. Nearly 100 compounds have been isolated from gastrodin. In 1978, gastrodin was isolated from the ethanolic extract of Rhizoma Gastrodiae for the first time [47, 48]. Through silica gel colum chromatography, 14 gastrodin compounds have been isolated and identified, included 8 phenolic components were fractionated: 4-hydroxybenzaldehyde (4-HBAL), 4-hydroxybenzyl alcohol (4-HBA), benzyl alcohol, bis-(4-hydroxyphenyl) methane, 4-(4^/^-hydroxybenzyloxy) benzyl methylether, 4-hydroxy-3-methoxybenzyl alcohol (vanillyl alcohol), 4-hydroxy-3-methoxybenzaldehyde (vanillin), and 4-hydroxy-3-methoxybenzoic acid (vanillic acid) [46, 49].

### Target population and data source

The target population of this study included inpatients whose primary diagnosis of hospital discharge was dizziness or vertigo according to the ICD-10 coding, treated between 1st January 2018 and 31st December 2018. In this study, data was collected from the Health Information System (HIS) of 131 hospitals around China, including 101 tertiary hospitals, 24 secondary hospitals, and 6 primary hospitals.

The specific inclusion criteria of the patients are:

1. Inpatient who diagnosed with dizziness or vertigo between 1st January 2018 and 31st December 2018;
2. Dizziness or vertigo was the principal diagnosis (ICD-10 code: H81 and R42);
3. Gastrodin injection alone or Extract of Ginkgo Biloba Leaves injection alone in clinical treatment.

The specific exclusion criteria of the patients are:

1. Patients who were younger than 18 years old or older than 75 years old;
2. Patients with cancers or pregnancy;
3. Patient record is incomplete.

Regarding patient inclusion, we selected the data in sequence. First, coding H81 and R42 with ICD-10 was used to filter and diagnose data containing H81 and R42 from the full data set to obtain the Per Protocol Set (PPS). Second, according to the subgroup design, the subgroup data was screened separately. The subgroup of dizziness and vertigo combined with cerebral infarction is a sub-data set filtered with the keyword “cerebral infarction” from PPS.

### Sampling

The included patients were divided into two samples: sample of dizziness or vertigo; sample of dizziness or vertigo complicated with cerebral infarction.

- Sample of dizziness or vertigo
  Patients were with the only principal diagnosis as dizziness or vertigo.
- Sample of dizziness or vertigo, with the complication of cerebral infarction
  Patients were with the principal diagnosis as dizziness or vertigo, and with another diagnosis as cerebral infarction.

### Treatment

All patients were divided into exposed group and control group. The exposed group was hospitalized with Gastrodin injection, and the control group was treated with Extract of Ginkgo Biloba Leaves injection. The outcomes and costs of Gastrodin injection and Extract of Ginkgo Biloba Leaves injection were compared.

- Gastrodin injection
  During hospitalization, the principal diagnosis was dizziness or vertigo and was prescribed Gastrodin injection, while Extract of Ginkgo Biloba Leaves injection and other Chinese medicine injections of dizziness or vertigo were not prescribed.
- Extract of Ginkgo Biloba Leaves injection
  During hospitalization, the principal diagnosis was dizziness or vertigo and was prescribed Extract of Ginkgo Biloba Leaves injection, while Gastrodin injection and other Chinese medicine injections of dizziness or vertigo were not prescribed.

Due to the lack of therapeutic dosage on the first page of the collected electronic health records, the therapeutic dosage could not be included in the analysis. The clinical experts we consulted indicated that clinicians usually chose therapeutic dosage according to patient status and disease progression in realistic practice. Thus, in this study, we compared the two groups (Gastrodin injection vs. Extract of Ginkgo Biloba Leaves injection), not the therapeutic dosage.

### Outcomes

This study’s outcome indicators included medical cost per hospitalization, hospitalization duration, effective rate, and cure rate, which were suggested by the clinical experts we consulted. Therapeutic effects are divided into death, ineffective, effective (turn for the better, cure), and others. Specifically, effective includes turn for the better and cure of clinical treatment. Cure refers to “the cure of each hospitalization”, and the cure rate refers to “the cure rate of each hospitalization”. The cure rate is based on the realistic observation and judgment of clinical doctors directly responsible for the patients, which is directly extracted from the original medical records in the HIS of the hospitals.

### Costs

In this study, the costs only considered the direct medical cost in the period of 1st January 2018 to 31st December 2018. Including total hospitalization costs, drug fees, laboratory fees, bed fees, operation fees, nursing fees, inspection fees, treatment fees, and other fees. Among them, the cost of drug includes target drug fee (Gastrodin injection or Extract of Ginkgo Biloba Leaves injection) and other drug fee. Cost data collected from HIS of 131 hospitals over the country. This study focused on medical costs per hospitalization because this data is the most reliable data to reflect treatments' economic effectiveness.

### Bias and elimination

Propensity score matching (PSM) is used to control the confounding factors and reduce the impact of confounding factors in evaluating intervention effects. In the study, logistic regression was used to score the tendency, mixed variables including region, hospital level, hospital department, age, and sex. PSM was achieved by Nearest Neighbor Matching (NNM) in the match it bag of R language, where the matching ratio is set to be 1:1.

### Statistical analysis

In this study, descriptive statistics analysis was conducted to describe basic characteristics, comparative analysis of outcome, and cost. Descriptive analysis was conducted as measurement data are described by median and quartile, and enumeration data are described by frequency and rate. The outcome, hospitalization cost and composition, percentage, and *P*-value of the two groups were also compared and analyzed by SPSS statistical software. *P* < 0.05 was considered statistically significant.

## Results

**Fig. 1:**
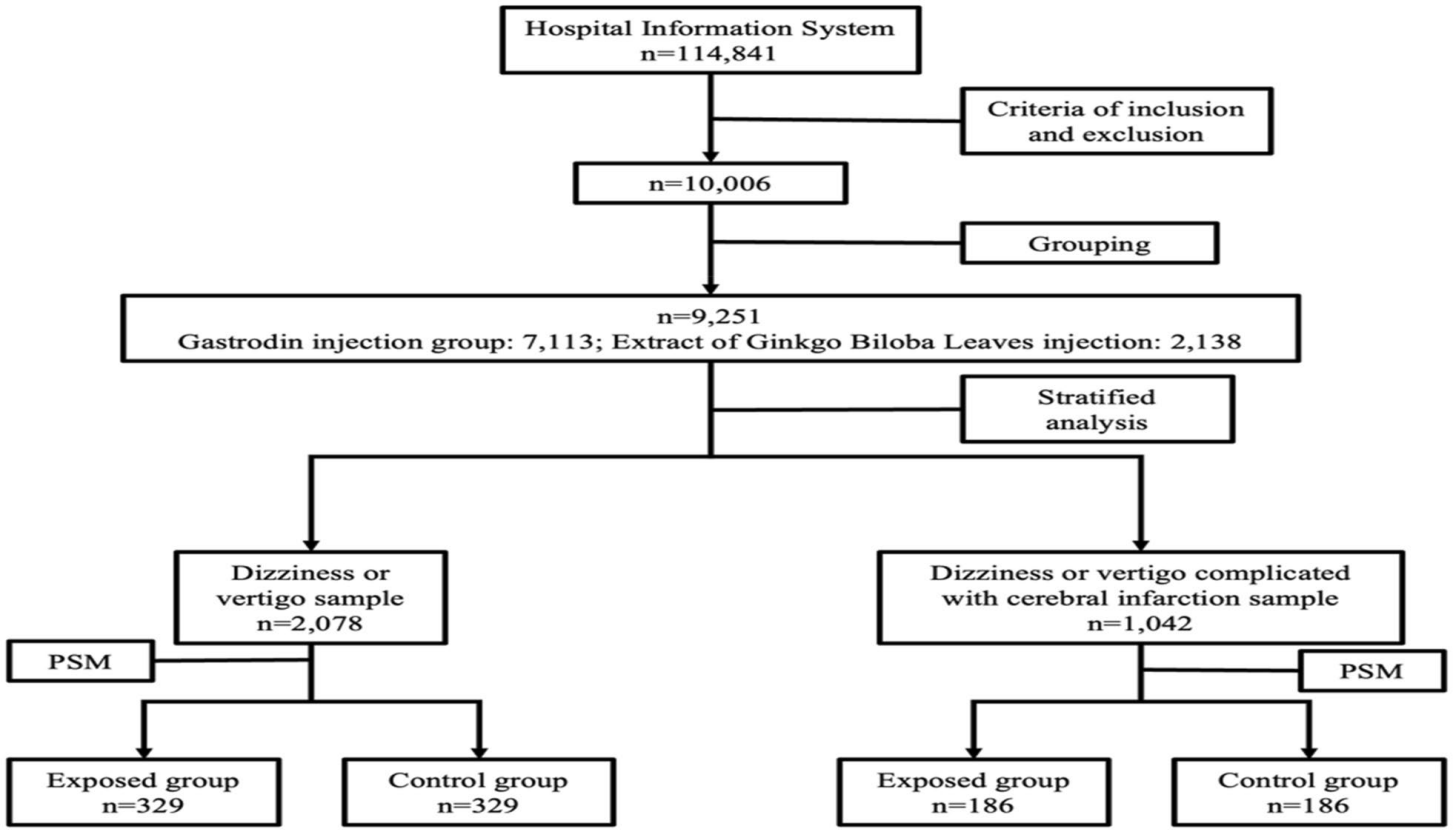
Flowchart of sampling

In this study, 9251 patients with dizziness or vertigo who met the inclusion and exclusion criteria were selected, including 7113 patients in the exposed group and 2138 patients in the control group. After stratified analysis, there were 2078 patients in the sample of dizziness or vertigo; and 1042 patients in the sample of dizziness or vertigo, with the complication of cerebral infarction. The flowchart of sampling was summarized in Fig. 1.

### Sample of dizziness or vertigo

#### Sample characteristics at baseline before PSM

As shown inTable 1, 1529 patients were included in Gastrodin injection treatment, while 528 patients were included in Extract of Ginkgo Biloba Leaves injection treatment. In these two groups, there were significant differences in region, hospital level, and hospital department (*P* < 0.001).

**Table 1:** Characteristics at baseline before PSM: sample of dizziness or vertigo

Variable	Total (N = 2057)	Gastrodin injection (N = 1529)	Extract of Ginkgo Biloba Leaves injection (N = 528)	P
Region, (n, %)				< 0.001
Eastern	1079 (52.5)	824 (53.9)	255 (48.3)	
Central	363 (17.6)	206 (13.5)	157 (29.7)	
Western	556 (27.0)	449 (29.4)	107 (20.3)	
Northeast	59 (2.9)	50 (3.3)	9 (1.7)	
Hospital level, (n, %)				< 0.001
Tertiary	1889 (91.8)	1384 (90.5)	505 (95.6)	
Secondary	139 (6.8)	122 (8.0)	17 (3.2)	
Primary	29 (1.4)	23 (1.5)	6 (1.1)	
Hospital department, (n, %)				< 0.001
Neurology	1492 (72.5)	1179 (77.1)	313 (59.3)	
E.N.T	146 (7.1)	37 (2.4)	109 (20.6)	
Cardiovascular medicine	29 (1.4)	23 (1.5)	6 (1.1)	
ICU	390 (19.0)	0	0	
Others	55 (45, 64)	290 (19.0)	100 (18.9)	
Age (y), (n, %)				0.674
[18, 45]	514 (25.0)	377 (24.7)	137 (25.9)	
[45,60]	750 (36.5)	555 (36.3)	195 (36.9)	
[60,75]	758 (36.8)	568 (37.1)	190 (36.0)	
≥ 75	35 (1.7)	29 (1.9)	6 (1.1)	
Gender (n, %)				0.959
Female	1235 (60.0)	917 (60.0)	318 (60.2)	
Male	822 (40.0)	612 (40.0)	210 (39.8)	

#### Sample characteristics at baseline after PSM

After PSM, the baseline was shown in Table 2. Gastrodin injection treatment group and Extract of Ginkgo Biloba Leaves injection treatment group finally included 329 patients, respectively. There was no difference at baseline between these two groups. In these two groups, it is mainly female, and the age is mainly 45 to 75 years old. There were about 60% patients come from eastern, about 97% patients come from tertiary hospitals and about 84% of them were treated in the Department of Neurology.

**Table 2:** Characteristics at baseline after PSM: sample of dizziness or vertigo

Variable	Gastrodin injection (N = 329)	Extract of Ginkgo Biloba Leaves injection (N = 329)	P	SMD
Region, (n, %)			0.79	0.080
Eastern	193 (58.7)	195 (59.3)		
Central	55 (16.7)	56 (17.0)		
Western	80 (24.3)	78 (23.7)		
Northeast	1 (0.3)	0 (0.0)		
Hospital level, (n, %)			1	< 0.001
Tertiary	320 (97.3)	320 (97.3)		
Secondary	7 (2.1)	7 (2.1)		
Primary	2 (0.6)	2 (0.6)		
Hospital department, (n, %)			0.909	0.058
Neurology	279 (84.8)	276 (83.9)		
E.N.T	7 (2.1)	10 (3.0)		
Cardiovascular medicine	1 (0.3)	1 (0.3)		
ICU	0	0		
Others	42 (12.8)	42 (12.8)		
Age (y), (n, %)			0.991	0.026
[18, 45]	63 (19.1)	60 (18.2)		
[45,60]	133 (40.4)	133 (40.4)		
[60,75]	128 (38.9)	131 (39.8)		
≥ 75	5 (1.5)	5 (1.5)		
Gender (n, %)			0.871	0.019
Female	211 (64.1)	208 (63.2)		
Male	118 (35.9)	121 (36.8)		

#### Comparison of outcomes

As shown in Table 3, compared with Extract of Extract of Ginkgo Biloba Leaves injection treatment, Gastrodin injection treatment had less hospitalization duration (7 (5, 10) versus 8 (6, 10)), but there was no any statistical significance (*P* = 0.080). There was a statistically significant difference between these two groups (*P* < 0.001) in terms of treatment effect. Specifically, the effective of Gastrodin injection treatment (100%) was higher than Extract of Ginkgo Biloba Leaves injection treatment (99.7%) with no significant difference (*P* = 1.000). However, the cure rate of Gastrodin injection treatment (42.9%) was higher than Extract of Ginkgo Biloba Leaves injection treatment (25.5%) with a significant difference (*P* < 0.05).

**Table 3:** Outcome comparison after PSM: sample of dizziness or vertigo

Variable	Gastrodin injection (N = 329)	Extract of Ginkgo Biloba Leaves injection (N = 329)	*P*
Hospitalization duration, M(Q_L_, Q_U_)	7 (5, 10)	8 (6, 10)	0.080
Hospitalization duration, (n, %)			0.022
0	0 (0.0)	3 (0.9)	
[1, 7]	169 (51.4)	139 (42.2)	
[8, 30]	159 (48.3)	185 (56.2)	
≥ 30	1 (0.3)	2 (0.6)	
Treatment effect, (n, %)			< 0.001
Death	0	0	
Ineffective	0	0	
Effective	329 (100)	328 (99.7)	1.000
Turn for the better	188 (57.1)	244 (74.2)	
Cure	141 (42.9)	84 (25.5)	< 0.05
Others	0 (0.0)	1 (0.3)	

#### Comparision of costs

As shown in Table 4, the total hospitalization cost of Gastrodin injection treatment (5530.00 (4054.00, 7702.00)) was significantly (*P* < 0.001) less than that of Extract of Ginkgo Biloba Leaves injection treatment (6672.00 (5057.00, 9023.00)). Except for the other drug fee and treatment fee, the sub cost items of Gastrodin injection treatment were significantly (*P* < 0.001) less than that of Extract of Ginkgo Biloba Leaves injection treatment. Besides, Gastrodin injection treatment's nursing fee was less than Extract of Ginkgo Biloba Leaves injection treatment, but without significant difference (*P* = 0.056).

**Table 4:** Cost comparison after PSM: sample of dizziness or vertigo

M (Q_L_, Q_U_)	Gastrodin injection (N = 329)	Extract of Ginkgo Biloba Leaves injection (N = 329)	*P*
Total hospitalization cost	5530.00 (4054.00, 7702.00)	6672.00 (5057.00, 9023.00)	< 0.001
Drug fees	1426.00 (808.00, 2446.00)	1858.00 (1121.00, 2710.00)	< 0.001
Target drug fee	326.16 (206.52, 464.67)	746.10 (483.80, 1040.17)	< 0.001
Other drug fee	1087.73 (507.11, 2059.70)	1032.40 (440.60, 1758.44)	0.169
Inspection fee	1532.00 (824.00, 2078.00)	2056.00 (1289.00, 2745.00)	< 0.001
Laboratory fee	950.00 (599.00, 1454.00)	1203.00 (781.00, 1533.00)	0.001
Treatment fee	573.00 (244.00, 1191.00)	348.00 (220.00, 752.00)	< 0.001
Bed fee	300.00 (200.00, 480.00)	364.00 (240.00, 550.00)	0.002
Nursing fee	146.00 (99.00, 220.00)	164.00 (108.00, 255.00)	0.056
Operation fee	0.00 (0.00, 0.00)	0.00 (0.00, 0.00)	< 0.001
Other fee	45.00 (1.00, 123.00)	63.00 (6.00, 515.00)	0.001
Daily average target drug fee	51.63 (34.42, 58.08)	96.76 (75.26, 120.95)	< 0.001
Daily average hospitalization fee	784.33 (544.30, 1089.33)	842.83 (645.90, 1102.71)	0.029
Per capita hospitalization cost, mean ± SD	6463.88 ± 3885.48	7165.25 ± 3275.85	< 0.001

### Sample of dizziness or vertigo, with the complication of cerebral infarction

#### Sample characteristics at baseline before PSM

Before PSM, 727 patients were included in Gastrodin injection treatment, while 315 patients included Extract of Ginkgo Biloba Leaves injection treatment. There were significant differences in region, hospital level, and age (see Table 5).

**Table 5:** Characteristics at baseline before PSM: sample of dizziness or vertigo, with the complication of cerebral infarction

Variable	Total (N = 1042)	Gastrodin injection (N = 727)	Extract of Ginkgo Biloba Leaves injection (N = 315)	P
Region, (n, %)				< 0.001
Eastern	427 (41.0)	323 (44.4)	104 (33.0)	
Central	305 (29.3)	136 (18.7)	169 (53.7)	
Western	228 (21.9)	205 (28.2)	23 (7.3)	
Northeast	82 (7.9)	63 (8.7)	19 (6.0)	
Hospital level, (n, %)				0.001
Tertiary	983 (94.3)	677 (93.1)	306 (97.1)	
Secondary	32 (3.1)	31 (4.3)	1 (0.3)	
Primary	27 (2.6)	19 (2.6)	8 (2.5)	
Hospital department, (n, %)				0.064
Neurology	776 (74.5)	541 (74.4)	235 (74.6)	
E.N.T	18 (1.7)	0	0	
Cardiovascular medicine	1 (0.1)	17 (2.3)	1 (0.3)	
ICU	247 (23.7)	1 (0.1)	0 (0.0)	
Others	64 (56, 69)	168 (23.1)	79 (25.1)	
Age (y), (n, %)				0.022
[18, 45]	35 (3.4)	19 (2.6)	16 (5.1)	
[45,60]	314 (30.1)	230 (31.6)	84 (26.7)	
[60,75]	657 (63.1)	448 (61.6)	209 (66.3)	
≥ 75	36 (3.5)	30 (4.1)	6 (1.9)	
Gender (n, %)				0.281
Female	510 (48.9)	364 (50.1)	146 (46.3)	
Male	532 (51.1)	363 (49.9)	169 (53.7)	

#### Sample characteristics at baseline after PSM

After PSM, the baseline was shown in Table 6. Gastrodin injection treatment group and Extract of Ginkgo Biloba Leaves injection treatment group finally included 186 patients, respectively. There was no difference at baseline between these two groups. In these two groups, it is mainly female (52.2%), and the age is mainly 60 to 75 years old. There were 48.4% patients come from eastern and 38.7% central, 99.5% patients come from tertiary hospitals and about 81.2% of them were treated in the Department of Neurology.

**Table 6:** Characteristics at baseline after PSM: sample of dizziness or vertigo, with the complication of cerebral infarction

Variable	Gastrodin injection (N = 186)	Extract of Ginkgo Biloba Leaves injection (N = 186)	P	SMD
Region, (n, %)			1	< 0.001
Eastern	90 (48.4)	90 (48.4)		
Central	72 (38.7)	72 (38.7)		
Western	15 (8.1)	15 (8.1)		
Northeast	9 (4.8)	9 (4.8)		
Hospital level, (n, %)			1	< 0.001
Tertiary	185 (99.5)	185 (99.5)		
Secondary	0	0		
Primary	1 (0.5)	1 (0.5)		
Hospital department, (n, %)			1	< 0.001
Neurology	151 (81.2)	151 (81.2)		
E.N.T	0	0		
Cardiovascular medicine	0	0		
ICU	0	0		
Others	35 (18.8)	35 (18.8)		
Age (y), (n, %)			1	< 0.001
[18, 45]	4 (2.2)	4 (2.2)		
[45,60]	55 (29.6)	55 (29.6)		
[60,75]	123 (66.1)	123 (66.1)		
≥ 75	4 (2.2)	4 (2.2)		
Gender (n, %)			1	< 0.001
Female	97 (52.2)	97 (52.2)		
Male	89 (47.8)	89 (47.8)		

#### Comparison of outcomes

As shown in Table 7, between Gastrodin injection treatment and Extract of Ginkgo Biloba Leaves injection treatment, there was no significant difference for hospitalization duration (*P* = 0.371), but a significant difference for the treatment effect (*P* = 0.009). Specifically, the effective of Gastrodin injection treatment (99.0%) was less than Extract of Ginkgo Biloba Leaves injection treatment (100%) with no significant difference (*P* = 0.499). However, the cure rate of Gastrodin injection treatment (32.3%) was higher than Extract of Ginkgo Biloba Leaves injection treatment (21.0%) with a significant difference (*P* < 0.05).

**Table 7:** Outcome comparison after PSM: sample of dizziness or vertigo, with the complication of cerebral infarction

Variable	Gastrodin injection (N = 186)	Extract of Ginkgo Biloba Leaves injection (N = 186)	*P*
Hospitalization duration, M(Q_L_, Q_U_)	9 (7, 12)	10 (7, 13)	0.371
Hospitalization duration, (n, %)			0.516
0	2 (1.1)	0 (0.0)	
[1, 7]	57 (30.6)	55 (29.6)	
[8, 30]	127 (68.3)	130 (69.9)	
≥ 30	0 (0.0)	1 (0.5)	
Treatment effect, (n, %)			0.009
Death	0	0	
Ineffective	0	0	
Effective	184 (99.0)	186 (100)	0.499
Turn for the better	124 (66.7)	147 (79.0)	
Cure	60 (32.3)	39 (21.0)	< 0.05
Others	2 (1.1)	0 (0.0)	

#### Comparision of costs

As shown in Table 8, the total hospitalization cost of Gastrodin injection treatment (7391.47 (5030.42, 10,654.81)) was less than that of Extract of Ginkgo Biloba Leaves injection treatment (8212.60 (5243.71, 12,633.69)) with no significant difference (*P* = 0.123). In terms of the drug fee, target drug fee, inspection fee, daily average target drug fee, and per capita hospitalization cost, Gastrodin injection group was significantly (*P* < 0.001) less than that of Extract of Ginkgo Biloba Leaves injection treatment.

**Table 8:** Cost comparison after PSM: sample of dizziness or vertigo, with the complication of cerebral infarction

M (Q_L_, Q_U_)	Gastrodin injection (N = 186)	Extract of Ginkgo Biloba Leaves injection (N = 186)	*P*
Total hospitalization cost	7391.47 (5030.42, 10,654.81)	8212.60 (5243.71, 12,633.69)	0.123
Drug fees	2671.15 (1501.84, 4144.48)	2883.27 (1881.75, 4982.69)	0.039
Target drug fee	361.41 (206.52, 516.30)	677.32 (387.04, 1064.36)	< 0.001
Other drug fee	2240.97 (1152.53, 3842.02)	2101.26 (1254.55, 4199.22)	0.661
Inspection fee	1635.00 (1054.25, 2206.66)	1926.00 (1124.50, 2880.00)	0.005
Laboratory fee	1209.50 (722.15, 1776.38)	1079.00 (825.35, 1910.65)	0.687
Treatment fee	566.33 (235.75, 1272.78)	403.00 (172.37, 874.44)	0.147
Bed fee	410.00 (250.00, 560.00)	420.00 (251.03, 599.88)	0.692
Nursing fee	222.19 (126.47, 347.91)	206.76 (141.69, 381.78)	0.712
Operation fee	0.00 (0.00, 0.00)	0.00 (0.00, 0.00)	0.761
Other fee	87.50 (20.50, 325.12)	75.60 (5.20, 312.00)	0.293
Daily average target drug fee	51.63 (25.82, 57.37)	85.83 (42.63, 106.22)	< 0.001
Daily average hospitalization fee	824.75 (609.41, 1120.93)	872.35 (670.06, 1212.33)	0.122
Per capita hospitalization cost, mean ± SD	9315.75 ± 9472.04	11,715.70 ± 14,086.39	< 0.001

## Discussion

In this study, we focused on the clinical and economic anlaysis of Gastrodin injection, comparing with Extract of Ginkgo Biloba Leaves injection, which is commonly used to treat dizziness or vertigo [50, 51]. We compared the Extract of Ginkgo Biloba Leaves injection with Gastrodin injection through clinical and economic evaluation outcome indicators such as medical cost of hospitalization, days of hospital stay, effective rate, and cure rate, to analyze the drug economy and effectiveness of Gastrodin injection in the real world. The data of this study was from real-world, not from controlled clinical trials. Real-world data is different from controlled clinical trial data, real-world evidence provides external validity and can provide evidence for the effectiveness and economic effects [52]. Real-world evidence can be used to complement data from controlled clinical trials to enable the generalization of clinical findings to a more inclusive and larger population, it can provide instructive information for clinical practice [53]. The correlations of gastrodin injection in the treatment of dizziness or vertigo were analyzed, and the differences of these two groups were found. So, this study’s results can provide valuable reference for the clinical practice of dizziness or vertigo.

Nevertheless, the use of real-world data is prone to pose selection bias challenges [54]. Therefore, to reduce the impact of confounding factors on intervention effect estimation, stratified analysis and PSM were used in this study. After PSM, the baseline situation between the exposed and control groups was more balanced and comparable than before, and some significant findings were generated that are worth further discussion below.

Regarding hospitalization duration, it found that there were no significant differences that of these two study groups in the sample of dizziness or vertigo and the sample of dizziness or vertigo complicated with cerebral infarction. In the past clinical study, it found that the combination of Gastrodin injection and conventional treatment had significant (*P* < 0.05) advantages in the improvement time compared with conventional treatment for the treatment of vertigo [55].

Regarding medical cost per hospitalization, this study found that the medical const per hospitalization of Gastrodin injection group was significantly (*P* < 0.001) lower than of Extract of Ginkgo Biloba Leaves injection group in the sample of dizziness or vertigo, but no significant differences that of these two study groups in the sample of dizziness or vertigo complicated with cerebral infarction. The economic advantage of Gastrodin injection for dizziness or vertigo patients can be proven in this study.

Although Gastrodin injection is effective in treating dizziness or vertigo [56], it also has some adverse drug reactions (ADRs) occasionally, such as dry mouth and nose, local pain, rash, and somnolence, etc. Based on two meta-analyses, few studies reported ADRs, while reported ADRs were mild and did not need special treatment [27, 28]. In a multi-center single-blind randomized controlled trial, of the 120 patients in the Gastrodin injection group, 10 had adverse reactions; the ADRs rate was 8.33% in the Gastrodin injection group and 10.83% in the control group without statistical significance (*P* = 0.538) [29]. However, most of the ADRs will return to normal after stopping the drug. Therefore, although the ADRs rate reported in the past studies is low and mild, Gastrodin injection safety still needs to be further clinical verification. It implies that patient-centered clinical medication needs more exploration.

Also, the strengths of this study are obvious. This study reflects the correlation of gastrodin injection in the treatment of dizziness or vertigo, and also shows that there are differences between Gastrodin injection and Extract of Ginkgo Biloba Leaves injection. Firstly, the actual clinical practice can be shown by using real-world data to conduct the clinical and economic evaluation of Gastrodin injection for dizziness or vertigo. Secondly, the data of this study were collected from 131 hospitals across the country, which covered the whole nation, so the findings have more generalizing significance for dizziness or vertigo treatment. Thirdly, there is still lacking studies to evaluate the clinical and economic benefit of the Gastrodin injection with the comparison of other drugs for patients in the actual clinical practice. Therefore, this study supplemented the evidence in this respect.

## Limitations and future research

However, this study has some limitations which can be addressed in future studies. First, this study recorded the realistic drug utilization data without the standardized dosage of different treatments in the real-world data of electronic health record data. Prospective desing of pragmatic controlled trial with a standardized therapeutic dosage in the future studies can generate more direct evidence of Gastrodin injection on dizziness and vertigo. Second, it is impossible to eliminate the influence of all confounding factors, such as Charlson Comorbidity Index (CCI), disease course, concomitant medication, cerebral infarction subclass, etc. Future studies can particularly explore the effects of Gastrodin Injection on dizziness and vertigo in specific disease areas. Third, this research only compared two naturally extracted medicines without considering conventional medicine. Thus, future studies can use conventional medicines as comparators to test the two naturally extracted medicines' clinical effects. Fourth, hospital policy, insurance policies, and pharmaceutical factories can influence drug utilization. However, in this research, we only focus on the personal patient level by using individual data of electronic medical records to test the clinical and economic effects of two naturally extracted medicines. The impacts of these contextual factors need further exploration in future studies. Last, the sample patients of this study only from inpatients, the findings can provide references for clinicians to optimize treatment options and for health insurance departments to make relevant policies. But may not reflect the whole disease populations without outpatients. Thus, future studies can include outpatients and inpatients for the comprehensive research.

## Conclusions

Gastrodin injection showed advantages for inpatients with dizziness or vertigo compared with Extract of Ginkgo Biloba Leaves injection. Future studies using prospective pragmatic controlled trials can test and explore more about the effects of Gastrodin injections on dizziness or vertigo.

## Supplementary Information

**Additional file 1:** STROBE Statement—Checklist of items that should be included in reports of ***cohort studies***.

## Data Availability

The data can be provided from corresponding authors upon reasonable request.

## Abbreviations

ADRs: Adverse drug reactions
HIS: Hospital Information System
PSM: Propensity score matching
4-HBAL: 4-Hydroxybenzaldehyde
4-HBA: 4-Hydroxybenzyl alcohol
PPS: Per Protocol Set
NNM: Nearest Neighbor Matching
CCI: Charlson Comorbidity Index
